# Bioprospecting endophytic fungi for antifeedants and larvicides and their enhancement by gamma irradiation

**DOI:** 10.1186/s13568-022-01461-3

**Published:** 2022-09-16

**Authors:** Magdia A. Hazaa, Magdy M. Shebl, El-Sayed R. El-Sayed, Samar R. Mahmoud, Abeer A. Khattab, Mahmoud M. Amer

**Affiliations:** 1 Biological Applications Department, Nuclear Research Center, Egyptian Atomic Energy Authority, Cairo, Egypt; 2 Plant Research Department, Nuclear Research Center, Egyptian Atomic Energy Authority, Cairo, Egypt; 3 Department of Botany and Microbiology Faculty of Science, Benha University, Benha, Qalubiya Governorate Egypt

**Keywords:** Antifeedant, Larvicidal, GC-MS analysis, Gamma radiation, Endophytic Fungi, *Spodoptera littoralis*

## Abstract

The search and discovery of new natural products with antifeedant and larvicidal potentials to mitigate harmful insects are scientific pressing issues in the modern agriculture. In this paper, the antifeedant and larvicidal potentials of 69 fungal isolates were screened against the Egyptian cotton leafworm *Spodoptera littoralis*. A total of 17 isolates showed the insecticidal potentials with three promising isolates. These strains were *Aspergillus sydowii*, *Lasiodiplodia theobromae*, and *Aspergillus flavus* isolated from *Ricinus communis* (bark), *Terminalia arjuna* (Bark), and *Psidium guajava* (twigs), respectively. The effect of gamma irradiation on the antifeedant and larvicidal activities of the three strains was investigated. Exposure of the fungal spores to 1000 Gy of gamma rays significantly intensified both the antifeedant and larvicidal potentials. To identify compounds responsible for these activities, extracts of the three strains were fractionated by thin layer chromatography. The nature of the separated compounds namely, Penitrem A, 1, 3, 5, 8- tetramethyl- 4, 6-diethyl- 7- [2- (methoxycarbonyl)ethyl] porphyrin (from *A. sydowii*), Penitrem A, 2, 7, 12, 17-Tetramethyl-3, 5:8, 10:13, 15:18, 20-tetrakis (2,2-dimethylpropano) porphyrin (from *A. flavus*), N,N-Diethyl-3-nitrobenzamide, and Diisooctyl-phthalate (from *L. theobromae*) were studied by GC-MS analysis. These findings recommend endophytic fungi as promising sources of novel natural compounds to mitigate harmful insects.

## Key points

- Three promising fungal endophytes with antifeedant and larvicidal activities were reported.
- The antifeedant and larvicidal activities were intensified following exposure of fungal spores to 1000 Gy gamma rays.
- Extracts of the three strains were separately fractionated by TLC then GC-MS was used to identify chemical constituents responsible for bioactivity.

## Introduction

There is a continuous need to search for new natural compounds to provide protection to important crops from pests and thereby ensuring a sustainable food production process (Jamiołkowska and Kopacki 2020). Besides, the pressing scientific and social necessities to develop safe, cheap, and eco-friendly alternatives to avoid hazards resulting from the excessive use of synthetic pesticides (Manu et al. 2021). Over the years, the use of phyto-chemicals as pesticides were extensively studied (Ali et al. 2021) with the result of the identification of several leads from a wide range of plant species (Walia et al. 2014). Although, plants proved to be a rich source of natural compounds for insect control, the very low yields and unsustainable supply were the main drawbacks (Molyneux et al. 2007). Recently, the scientific community was directed to exploit microorganisms in this regard as sustainable sources for obtaining bioactive molecules with insecticidal potential (Grabka et al. 2022). In particular, fungal populations had several advantages including the cheap and simple growth and metabolism, tolerance to improvement and modification which rendered fungi the most robust strategy (Hussein et al. 2022).

Nowadays, the bioprospection of fungal endophytes for new compounds to control insect growth is gaining more attention (Grabka et al. 2022). Generally, these endophytes proved to be excellent and sustainable sources of many bioactive compounds with potential applications in pharmaceutical (Golinska et al. 2015), agriculture, and food industry (Sridhar 2019). All plant species have their own endophytes within tissues (Wang et al. 2016), as a result of their coevolutionary processes (Petrini et al. 1992; Ismaiel et al. 2017). In this regard, they can ensure the host plant survival by producing growth promoting metabolites, aiding the plant nutrient assimilation, and producing pest and insect repellents (Baron and Rigobelo 2022). Thus, the possibilities to find new compounds to control insect such endophytes are tremendous. Furthermore, their high metabolic activity (El-Sayed 2021) will ensure their excellence as the ideal biotechnological agent in this regard. For all of these reasons, we have already described the isolation of 69 fungal endophytes and the evaluation of their antimicrobial, anticancer and antioxidant activities (El-Sayed et al. 2022a).

As a part of our continuing research work in this concern, the current work was directed to unlock the untapped potential of the endophytes isolated from different plant species in Egypt as antifeedants and larvicides against the cotton leafworm *Spodoptera littoralis* (Bosid). This insect is considered as one of the most destructive agricultural lepidopterans with a resistance to several chemical insecticides (Hazaa et al. 2021). It attacks several economically significant crops belonging including crucifers, legumes, deciduous fruit trees, and grasses (El-Aswad et al. 2004). For example, it causes severe damage on cotton by feeding on the fruiting points, leaves, flower buds. As such, it’s larvae can bore into the tomato fruit rending it unsuitable for consumption (Khan and Ahmad 2015). In this paper, 69 fungal species were screened for the presence of antifeedant and larvicidal compounds in their cultures. Furthermore, the effect of gamma irradiation on the antifeedant and larvicidal properties of the fungal culture extracts was also adopted. Extracts from fungal cultures were fractionated by TLC then identified by GC-MS analysis.

## Materials and methods

### Endophytic fungi

Endophytic fungal isolates from our previous study (El-Sayed et al. 2022a) were used in this study to test their antifeedant and larvicidal potentials. Among the 69 fungal isolates, *Aspergillus sydowii* (an endophyte of *Ricinus communis*), *Aspergillus flavus* (an endophyte of *Psidium guajava*), and *Lasiodiplodia theobromae* (an endophyte of *Terminalia arjuna*) were selected according to our screening program (mentioned later). *A. sydowii* and *A. flavus* were previously identified (El-Sayed et al. 2022a). Identification of *Lasiodiplodia theobromae* was accomplished by colony morphology, growth characteristics, and molecular characterization. Morphological identification was performed by studying the colony on Czapek’s-yeast autolysate agar according to Moubasher (1993). Molecular characterization was performed according to the method by White et al. (1990) using PCR amplifed ITS1-5.8 S-ITS2 rRNA-gene. In brief, DNA of the fungal strains were extracted and sequenced by Solgent Company (Daejeon, South Korea). Sequences of the two strains were submitted to the GenBank and accession numbers were received. Finally, sequences were analyzed using the online tool (http://www.ncbi.nlm.nih.gov/) BLAST and the software BioEdit (version 7.0.1). Statistical tests for branch support were performed by approximate likelihood-ratio test using molbiol-tools/ Phylogeny (Phylogeny.fr) A neighbor-joining tree with the maximum-likelihood for each fungal strain were constructed using MEGA software version 6.0.

The three fungal endophytes were then deposited under numbers AUMC14506, AUMC14507, and AUMC14508 in the Culture Collection of Assiut University Mycological Center (AUMC), Egypt. Fungal strains were stored in the form of a suspension of spores and mycelia in 15% glycerol at − 4 °C.

### Test insect

*Spodoptera littoralis* (Boisduval 1833) (Lepidoptera: Noctuidae) cultures were initiated from egg-masses collected from an infested local field cultivated with cotton at Qualubia Governorate, Egypt. Confirmation of *S. littoralis* was performed by morphological identification (Blair 1974). The collected egg-masses were then surface sterilized with formalin vapor. The freshly hatched larvae were fed on castor oil (*Ricinus communis*) plant freshly collected leaves and kept in a 1600 cc glass jars. All jars were covered with muslin cloth and kept at 25℃ and 60–70% R. H. Finally, the 3rd instar larvae were collected and used in the screening program.

### Cultivation conditions

Spores of *A. sydowii* 14506, *L. theobromae* AUMC14508, and *A. flavus* AUMC14507 and were separately harvested from 7-days old culture. The final concentration of spores was 10^6^ mL^− 1^. Under aseptic conditions, 1 mL suspension was added to 50 mL (pH 6.0) sterilized Potato-dextrose broth in a 250 Erlenmeyer flask. The inoculated flasks for the three fungal strains incubated for 7 days at 30℃.

### Preparation of the fungal culture extracts

After incubation, culture filtrate for each fungal strain was filtered over Whatman No. 1 filter paper. A portion of the cell-free filtrate was kept for the evaluation process. The other portion was extracted with methylene chloride (1 : 1, thrice, on an equal volume basis) and the organic layer was filtered over anhydrous sodium sulphate. The filtered organic layer was evaporated using a rotary evaporator (IKA, RV10, Germany) under reduced pressure at 40 °C and the final dry film was dissolved in methanol. Finally, the concentrated extract was used for evaluation.

### Screening endophytic fungi for their antifeedant and larvicidal activities

#### Antifeedant bioassay

The antifeedant potential of the cell-free filtrate and the methylene chloride extract separated from the cultures of all the isolated endophytes was investigated using the leaf disc bioassay according to Duraipandiyan et al. (2011). Fresh castor oil leaf discs (4 cm diameter) were punched using a cork borer and dipped in either cell-free filtrate, methylene chloride extract, or methanol (control) and let to air-dry. To avoid early drying of the discs, it was placed in Petri dishes containing wet filter paper. Finally, the 3rd instar larvae of *S. littoralis* were introduced into Petri dishes containing the respective leaf discs. Each treatment was replicated five times with ten larvae in each replicate. The antifeedant activity was measured according to Thyagaraja and Rani (2019) in terms of reduction in the weight gain treated larvae as compared to the weight gain in control larvae after feeding for 48 h using the following formula:

Antifeedant activity (%) = [(Weight gain in control - Weight gain in treated)/(Weight gain in control)] × 100.

#### Larvicidal bioassay

The larvicidal potential of cell-free filtrate and methylene chloride extract of cultures from all the fungal isolates were performed according to WHO protocol (2005) using leaf dipping method (Hazaa et al. 2021). Fresh castor leaves were dipped in either cell-free filtrate, methylene chloride extract, methanol (control) and let to air-dry. To avoid early drying of the discs, it was placed in Petri dishes containing wet filter paper. The 3rd instar larvae of *S. littoralis* were introduced into Petri dishes containing the respective leaves and fed for 24 h, then maintained on untreated fresh leaves changed every 24 h. Five replicates were maintained for each treatment with ten larvae per replicate. The dead larvae were identified by the absence of movement and their subsequent change in color. Larval mortality was recorded every day for 14 days of treatment and percent of the cumulative mortality was estimated.

### **Effect of gamma irradiation on the antifeedant and larvicidal activities**

Spore suspensions of *A. sydowii*, *A. flavus* and *L. theobromae* were prepared. Every suspension was exposed to several doses of gamma rays in the range 250–4000 Gy. Gamma chamber (^60^Co, MC20, Russia) was used with the dose rate 432.80 Gy h^− 1^. Then, 1 mL of the irradiated suspensions for every fungal strain was used in fungal cultivation and preparation of the methylene chloride extract and evaluation of the antifeedant and larvicidal activities, as described earlier. Moreover, survival rate was estimated for each fungal strain as follows: 100 µL of the irradiated suspension was spread on potato-dextrose solid medium and incubated at 30 °C for 5 days. The survived colonies were counted and the number of colonies obtained from the control culture (non-irradiated) was considered to be 100% survival.

### Analytical methods

#### Estimation of dry biomass

After 7 days of the incubation period, the inoculated flasks were filtered through pre-weighted Whatman no.1 filter papers. The collected fresh biomass from each flask was dried at 50 °C in a hot air oven to a constant weight.

### Fractionation of the fungal extracts by thin layer chromatography (TLC)

The methylene chloride extracts of the three fungal strains was subjected to TLC on thin layer plates (0.25-mm GF-254, Loba Chemie Pvt. Ltd., Mumbai, India). A solvent mixture composed of 9:1 chloroform: methanol (v/v) was used. The developed plates were air-dried and examined under shortwave (245 nm) and longwave (365 nm) UV lamp. Consequently, the separated bands from each fungal extract lane were carefully removed by scraping off the silica gel and eluted with methanol. Each band separated from the TLC plates was tested for their antifeedant and larvicidal potentials (as described earlier).

### GC-MS analysis

Active bands from TLC were subjected to GC-MS analysis using a Thermo Scientific, Trace GCUltra/ISQ Single Quadrupole MS, TG-5MS fused silica capillary (30 m, 0.251 mm, 0.1 mm film thickness) column. The electron ionization system was of energy equal to 70 eV. Helium gas at a constant flow rate of 1 mL/min was used as the carrier. The MS transfer line and the injector temperature were set at 280℃. Identification of the compounds was performed based on the comparison of their mass spectra and relative retention times with those of the WILLY, NIST library data of the GC-MS system.

### Statistical analysis

Results were expressed as the mean ± standard deviation calculated from five replicate measurements from two independent experiment. Analysis of variance (ANOVA) followed by Dunken’s test were used to evaluate the significant significance (at 95% confidence intervals) using SPSS software (v. 22, IBM, NY).

## Results

### Endophytes’ screening for their antifeedant and larvicidal potentials

**Fig. 1:**
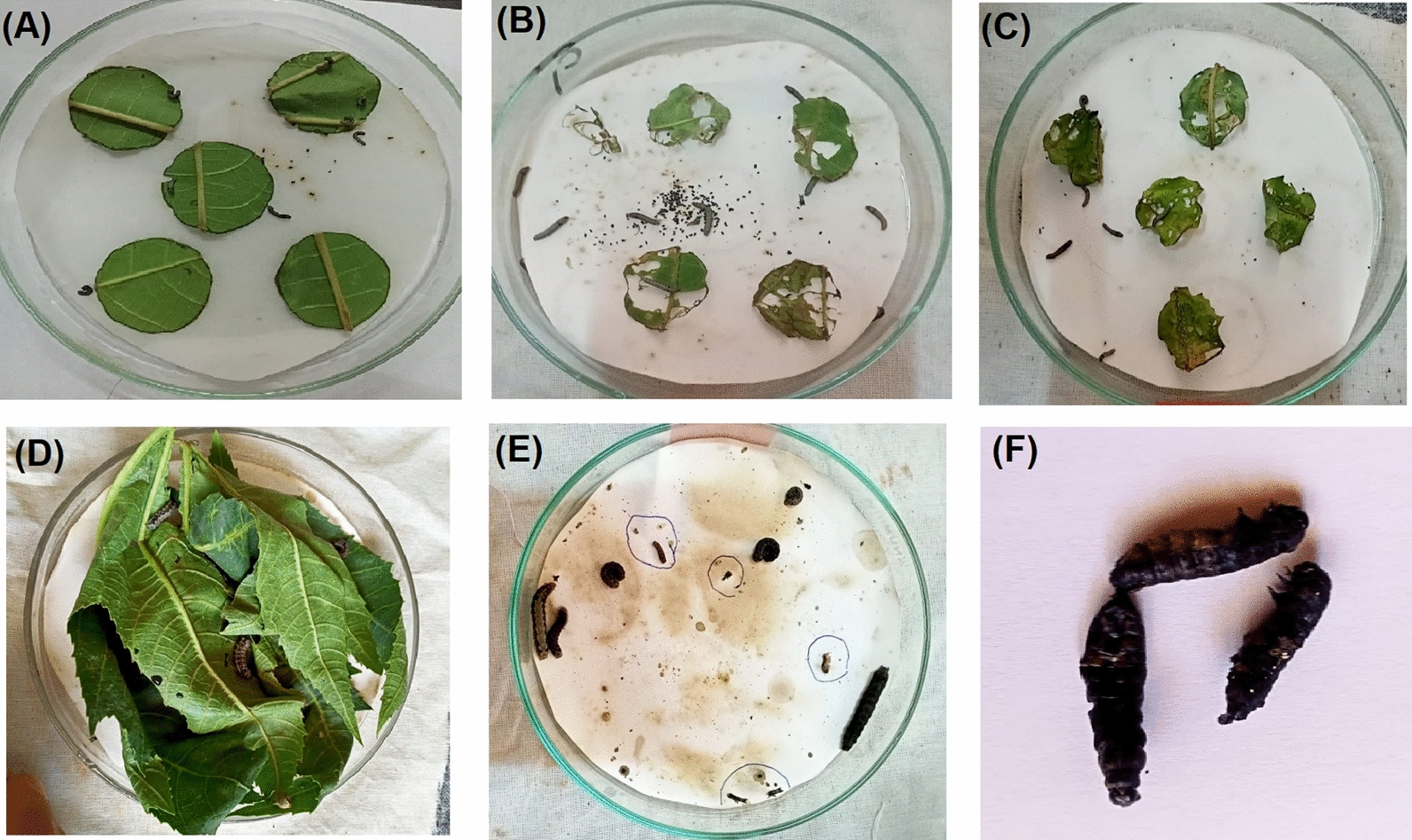
Photographs of the 3rd instar *Spodoptera littoralis* larvae feeding on castor leaves. **A** Leaf discs used in antifeedant activity evaluation after treatment. **B** Control untreated leaf discs with larvae after 48 h. **C** Treated discs with larvae fed after 24 h. **D** Treated castor leaves used in larvicidal activity evaluation with larvae fed at 0 h. **E, F** Dead larvae after fed on treated leaves

Fungi (69 endophytic isolates) were separately grown in PD broth at 30℃ for 7 days. Fungal cultures were filtered and a portion was extracted by methylene chloride. The cell-free filtrate and the methylene chloride extract were separately tested for their insecticidal activities against the cotton leafworm *Spodoptera littoralis*. Screening profile (Table 1; Fig. 1) of the 69 isolates revealed the presence of 17 positive isolates with larvicidal potential and 3 with antifeedant potential. Table 1 presented the preliminary morphological identification of the 17 isolates to the genus level. Among them, three isolates named Bb2, Mc1, and Db1 showed recorded the highest values of both the evaluated activities. Moreover, the recorded data (Table 1) indicated the superiority of the methylene chloride extract against the cell-free filtrate in all the tested activities. Accordingly, the three fungal isolates Bb2, Mc1, and Db1 were selected for identification and chemical characterization of their methylene chloride extracts.

**Table 1:** Host plants, isolated endophytic fungal genera, antifeedant activity (%), and cumulative mortality (%) of their cell-free filtrate and methylene chloride extract

Host plant	Plant part	Fungal genera	Code no.	Dry biomass (g L^− 1^)	Antifeedant activity (%)		Cumulative mortality (%)	
					cell-free filtrate	Methylene chloride	cell-free filtrate	Methylene chloride
*Ricinus communis*	Bark	*Aspergillus*	Bb2	8.76 ± 0.86^de^	28.57 ± 1.76^b^	46.52 ± 3.32^b^	38.76 ± 1.31^c^	56.66 ± 2.77^b^
	Twig	*Alternaria*	BC1	10.22 ± 0.67^bc^	–	–	14.21 ± 2.07^f^	36.66 ± 1.42^ef^
	Twig	*Trichoderma*	BC2	9.37 ± 0.76^cde^	–	–	21.65 ± 1.67^e^	44.55 ± 2.31^d^
	Bark	*Fusarium*	Bb3	11.06 ± 0.56^ab^	–	–	13.33 ± 2.43^f^	36.66 ± 2.43^ef^
*Hibiscus rose-sinensis*	Twig	*Aspergillus*	Cc1	8.76 ± 0.86^de^	–	–	30.67 ± 3.82^d^	23.33 ± 1.09^ g^
	Twig	*Alternaria*	Cc3	10.19 ± 0.69^bc^	–	–	30.21 ± 1.76^d^	36.55 ± 4.21^ef^
	Leaf	*Chaetomium*	Cb1	9.48 ± 0.58^cde^	–	–	39.98 ± 1.50^c^	33.33 ± 2.77^f^
*Terminalia arjuna*	Bark	*Lasiodiplodia*	Mc1	11.54 ± 0.17^ab^	37.94 ± 1.33^a^	52.56 ± 1.34^a^	59.44 ± 1.38^a^	63.33 ± 3.76^a^
	Leaf	*Alternaria*	Ma1	10.82 ± 0.82^bc^	–	–	20.13 ± 2.21^e^	26.46 ± 1.32^ g^
	Leaf	*Trichoderma*	Ma2	9.67 ± 0.38^cde^	–	–	23.33 ± 3.67^e^	21.65 ± 1.11^ h^
*Psidium guajava*	Twig	*Aspergillus*	Db1	8.78 ± 0.43^de^	21.57 ± 1.88^c^	41.89 ± 2.78^c^	44.67 ± 1.33^b^	50.11 ± 3.21^c^
	Bark	*Acremonium*	Db4	12.68 ± 0.62^a^	–	–	11.52 ± 3.71^f^	40.65 ± 2.12^de^
*Malus domestica*	Leaf	*Alternaria*	Ea2	10.93 ± 0.27^bc^	–	–	13.72 ± 4.58^f^	20.82 ± 1.44^ h^
	Bark	*Aspergillus*	Ea2	9.87 ± 0.17^cde^	–	–	23.67 ± 1.51^e^	43.33 ± 1.32^d^
*Citrus medica*	Bark	*Fusarium*	Fb2	10.11 ± 0.57^bc^	–	–	16.89 ± 2.43^f^	26.66 ± 1.54^ g^
	Bark	*Alternaria*	Fb3	8.89 ± 0.81^de^	–	–	7.76 ± 1.28^ g^	10.62 ± 0.98^i^
*Azadirachta indica*	Leaf	*Alternaria*	Aa2	9.69 ± 0.86^cde^	–	–	22.75 ± 3.17^e^	36.66 ± 2.41^ef^

PD broth was used for endophytic fungi cultivation. Static fungal cultures were incubated for 7 days at 30℃. Calculated mean is for five replicate measurements from two independent experiments ± SD, ^a−i^ means with different superscripts in the same column are considered statistically different (LSD test, *P* ≤ 0.05)

### Morphological and molecular identification

**Fig. 2:**
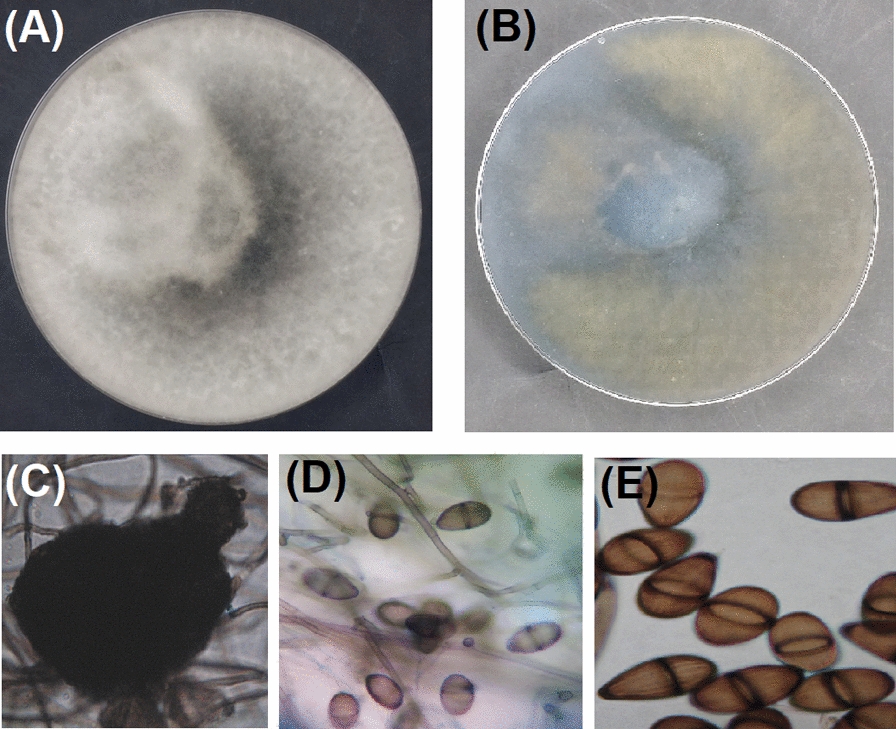
Morphological characteristics of *L. theobromae* AUMC14508. **A** Front colony view. **B** Reverse colony view. **C–E** Microscopic appearance

**Fig. 3:**
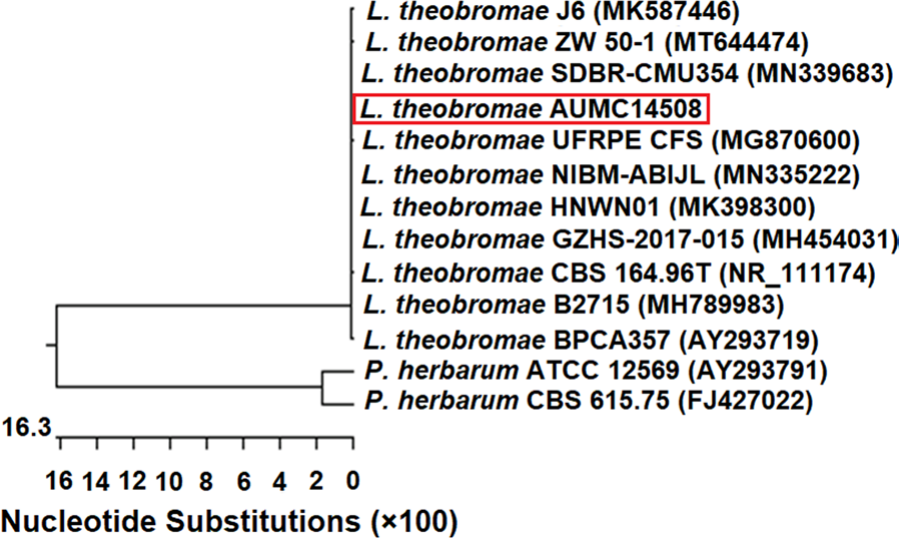
Phylogenetic tree of the fungal isolate *L. theobromae* AUMC14508 and other closely related strains, based on the ITS15.8 S rRNAITS2 rDNA sequences

The two isolates Bb2 and Db1 were previously identified in our previous study as *A. sydowii* and *A. flavus*, respectively. Meanwhile, Fig. 2 presented the colony morphology of *Lasiodiplodia theobromae* grown on CYA-agar plates. *L. theobromae* had fast growing, dark colored colony at 25 °C after 10 days. Microscopic examination further showed dark colored flask-shaped pycnidium which contains asexual conidiospores. Sequences of *L. theobromae* obtained from molecular characterization were deposited in the GenBank under number MW092908, then analyzed and a phylogenetic tree was built. Figure 3 presented the constructed phylogenetic tree confirming that *L. theobromae* AUMC14508 showed 100% identity with strains of *L. theobromae* including the Type strain CBS16496T (NR_111174), SDBR-CMU354 (MN339683), UFRPE CFS (MG870600), and NIBM-ABIJL (MN335222). Strains of *Phoma herbarum* are included as outgroup fungal species in the tree (Fig. 3).

### Improving the antifeedant and larvicidal activities by gamma irradiation

Spores of *A. sydowii*, *A. flavus* and *L. theobromae* were separately subject to gamma radiation at several doses to study the effect of exposure to gamma rays on the antifeedant and larvicidal activities of the three strains. Generally, the remarkable feature of the obtained results in Tables 2 and 3, and 4 is the dose-dependent manner of the influence of irradiation on both the survival rate and fungal growth of *A. sydowii*, *A. flavus* and *L. theobromae*. Tables 2 and 3, and 4 further indicated that the most proper dose for achieving the highest values of the antifeedant and larvicidal potentials for the three strains was at 1000 Gy. At this dose of gamma irradiation, significant differences in the recorded values of both the antifeedant and larvicidal activities as compared to the control non-irradiated cultures were observed (Tables 2 and 3, and 4).

**Table 2:** Effect of different doses of gamma irradiation on survival (%), dry biomass (g L^− 1^), antifeedant activity (%), and cumulative mortality (%) of the 3rd instar *Spodoptera littoralis* larvae feeding on castor leaves treated with methylene chloride extract of *A. sydowii* AUMC14506

Gamma irradiation dose (Gy)	Survival (%)	Dry biomass (g L^− 1^)	Antifeedant activity (%)	Cumulative mortality (%)
0.00 (C)	100 ± 0.00^a^	8.66 ± 0.72^a^	46.61 ± 2.52^c^	56.84 ± 2.12^c^
250	98.45 ± 1.65^a^	8.01 ± 0.32^a^	48.91 ± 2.67^c^	59.51 ± 1.76^c^
500	86.56 ± 1.21^b^	7.43 ± 0.57^ab^	56.41 ± 1.85^b^	66.52 ± 3.21^b^
1000	65.77 ± 2.55^c^	3.21 ± 0.22^b^	62.69 ± 2.66^a^	72.93 ± 1.77^a^
2000	28.18 ± 1.53^d^	1.92 ± 0.71^b^	5.22 ± 0.44^d^	10.41 ± 1.01^d^
4000	0.00 ± 0.00^e^	0.00 ± 0.00^c^	0.00 ± 0.00^e^	0.00 ± 0.00^e^

PD broth was used for cultivation. Static fungal cultures were incubated for 7 days at 30℃. Calculated mean is for five replicate measurements from two independent experiments ± SD, ^a−e^ means with different superscripts in the same column are considered statistically different (LSD test, *P* ≤ 0.05)

**Table 3:** Effect of different doses of gamma irradiation on survival (%), dry biomass (g L^− 1^), antifeedant activity (%), and cumulative mortality (%) of the 3rd instar *Spodoptera littoralis* larvae feeding on castor leaves treated with methylene chloride extract of *A. flavus* AUMC14507

Gamma irradiation dose (Gy)	Survival (%)	Dry biomass (g L^− 1^)	Antifeedant activity (%)	Cumulative mortality (%)
0.00 (C)	100 ± 0.00^a^	8.69 ± 0.17^a^	41.89 ± 2.78^c^	50.11 ± 3.21^c^
250	97.21 ± 2.01^a^	7.31 ± 0.42^a^	44.56 ± 1.77^c^	53.65 ± 1.61^c^
500	85.44 ± 4.65^b^	6.55 ± 0.81^ab^	53.22 ± 1.34^b^	62.65 ± 2.98^b^
1000	67.19 ± 3.11^c^	3.07 ± 0.13^b^	60.55 ± 2.31^a^	70.59 ± 1.0177^a^
2000	32.87 ± 2.98^d^	2.18 ± 0.05^b^	4.89 ± 0.96^d^	8.39 ± 0.78^d^
4000	0.00 ± 0.00^e^	0.00 ± 0.00^c^	0.00 ± 0.00^e^	0.00 ± 0.00^e^

PD broth was used for cultivation. Static fungal cultures were incubated for 7 days at 30℃. Calculated mean is for five replicate measurements from two independent experiments ± SD, ^a−f^ means with different superscripts in the same column are considered statistically different (LSD test, *P* ≤ 0.05)

**Table 4:** Effect of different doses of gamma irradiation on survival (%), dry biomass (g L^− 1^), antifeedant activity (%), and cumulative mortality (%) of the 3rd instar *Spodoptera littoralis* larvae feeding on castor leaves treated with methylene chloride extract of *L. theobromae* AUMC14508

Gamma irradiation dose (Gy)	Survival (%)	Dry biomass (g L^− 1^)	Antifeedant activity (%)	Cumulative mortality (%)
0.00 (C)	100 ± 0.00^a^	11.61 ± 0.52^a^	52.63 ± 3.21^d^	63.51 ± 2.41^d^
250	81.82 ± 2.71^b^	10.21 ± 0.41^a^	58.67 ± 3.41^c^	69.43 ± 3.22^c^
500	72.43 ± 3.52^c^	6.02 ± 0.89^b^	64.18 ± 2.76^b^	76.11 ± 2.51^b^
1000	45.51 ± 1.90^d^	4.65 ± 0.22^b^	71.43 ± 1.57^a^	79.24 ± 4.08^a^
2000	6.91 ± 1.88^e^	1.33 ± 0.86^c^	21.56 ± 2.33^e^	38.53 ± 2.55^e^
4000	0.00 ± 0.00^f^	0.00 ± 0.00^d^	0.00 ± 0.00^f^	0.00 ± 0.00^f^

PD broth was used for cultivation. Static fungal cultures were incubated for 7 days at 30℃. Calculated mean is for five replicate measurements from two independent experiments ± SD, ^a−f^ means with different superscripts in the same column are considered statistically different (LSD test, *P* ≤ 0.05)

### Separation of active constituents by TLC chromatography and identification by GC–MS

**Fig. 4:**
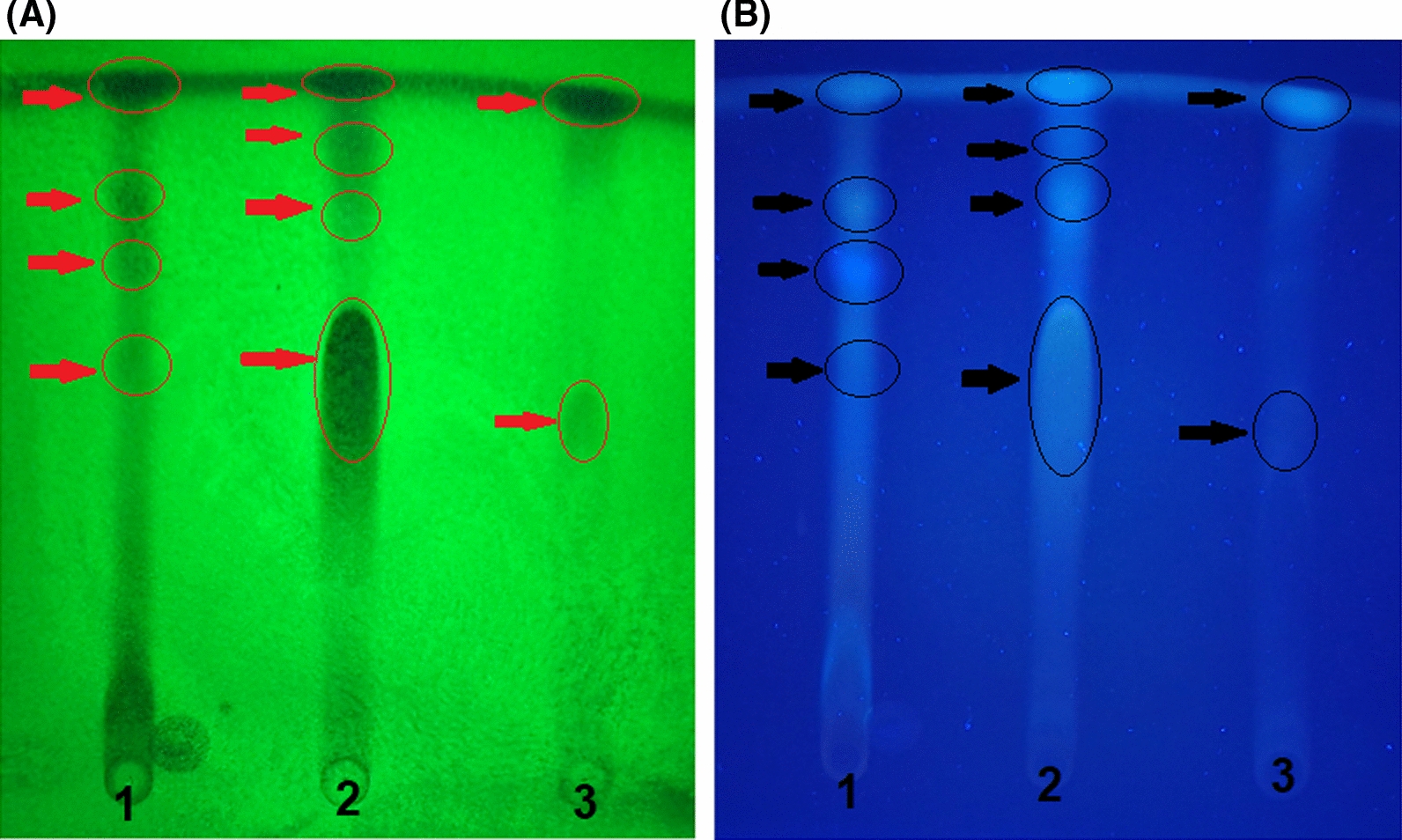
TLC chromatograms of the methylene chloride extract of *A. sydowii* AUMC14506 **(Lane 1)**, *A. flavus* AUMC14507 **(Lane 2)**, and *L. theobromae* AUMC14508 **(Lane 3)** under UV-light 254 nm **(A)** and UV-light 365 nm **(B)**

The methylene chloride extract was subjected to thin layer chromatography (TLC). Figure 4 presented the separated bands from each fungal extract visualized under 245 nm (Fig. 4 A) and 365 nm (Fig. 4B) UV light. All the separated bands were tested for their antifeedant and larvicidal activity to detect the active fractions. The active bands were band No. 2 for *A. sydowii*, band No. 2 for *A. flavus*, and band No. 1 for *L. theobromae*. The three bands were subjected to GC Mass to identify compounds responsible for the antifeedant and larvicidal activities.

**Fig. 5:**
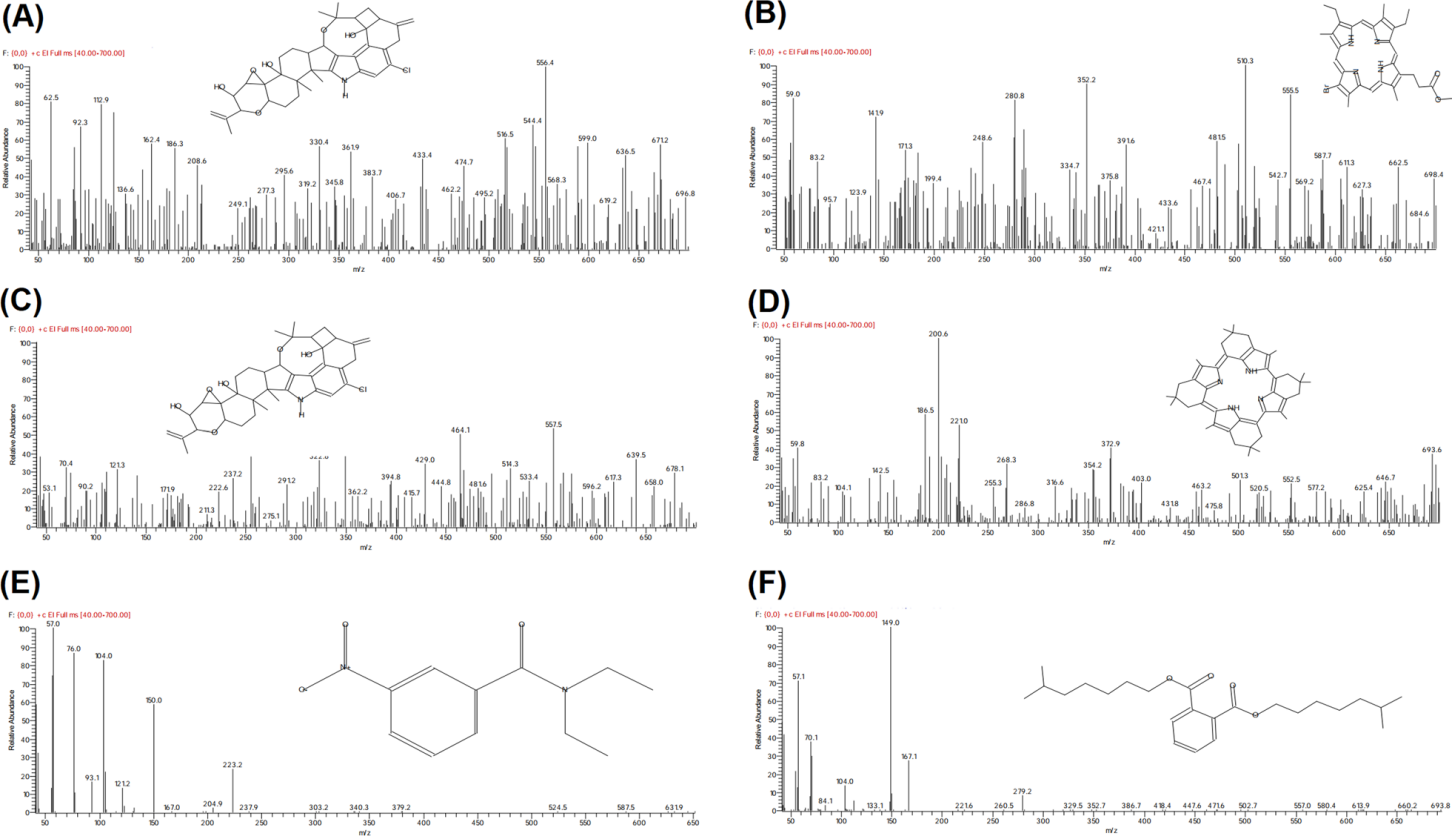
Chromatograms of the identified compounds with their structures. **A, B** From *A. sydowii* AUMC14506 cultures. **C, D** From *A. flavus* AUMC14507 cultures. **E, F** From *L. theobromae* AUMC14508 cultures

GC-MS analysis of the separated bands revealed the presence of 6 different compounds. The identification was accomplished using computer search user-generated reference libraries, incorporating mass spectra. Structures of the detected compounds, molecular weights and molecular formulas were listed in Table 5. The identified compounds for *A. sydowii* were PENITREM A (Fig. 5A) and 1,3,5,8-tetramethyl-4,6-diethyl-7-[2-(methoxycarbonyl)ethyl]porphyrin (Fig. 5B). In the case of *A. flavus*, the identified compounds were PENITREM A (Fig. 5c) and 2,7,12,17-Tetramethyl-3,5:8,10:13,15:18,20-tetrakis(2,2-dimethylpropano)porphyrin (Fig. 5D). In the case of *L. theobromae*, the identified compounds were N-Diethyl-3-nitrobenzamide (Fig. 5E) and Diisooctyl-phthalate (Fig. 5F).

**Table 5:** GC-MS analysis of the active fractions separated from TLC chromatography

Fungal strain	Molecular weight	Molecular formula	Detected compounds
*A. sydowii* AUMC14506	633	C_37_H_44_ClNO_6_	PENITREM A
	586	C_32_H_35_BrN_4_O_2_	1,3,5,8-tetramethyl-4,6-diethyl-7-[2-(methoxycarbonyl)ethyl]porphyrin
*A. flavus* AUMC14507	633	C_37_H_44_ClNO_6_	PENITREM A
	638	C_44_H_54_N_4_	2,7,12,17-Tetramethyl-3,5:8,10:13,15:18,20-tetrakis(2,2-dimethylpropano)porphyrin
*L. theobromae* AUMC14508	222	C_11_H_14_N_2_O_3_	N,N-Diethyl-3-nitrobenzamide
	390	C_24_H_38_O_4_	Diisooctyl-phthalate

## Discussion

Nowadays, fungal endophytes are considered an emerging source for several bioactive metabolites (Mousa et al. 2021; Hussein et al. 2022) including insecticidal that will be exploited in many agricultural applications and food industries. In this manner, the aim of the presented research lies in an underestimated and unexploited research subject: antifeedants and larvicides from endophytic fungal isolates of some plants in Egypt. Our results confirmed that three isolates named Bb2, Mc1, and Db1, showed the highest values of both the evaluated activities. As such, the obtained results indicated the superiority of the methylene chloride extract over the cell-free filtrate in the tested antifeedant and larvicidal bioactivities. The two fungal isolates Bb2 and Db1 were identified in our previous study as *Aspergillus sydowii* AUMC14506 and *Aspergillus flavus* AUMC14507, respectively (El-Sayed et al. 2022a). Meanwhile, morphological characteristics of the third fungal strain Mc1 were identical with those concerning the identification of *Lasiodiplodia theobromae* (Moubasher 1993). Furthermore, the molecular characterization of the Mc1 strain showed high conformity with *Lasiodiplodia theobromae* related strains. *Lasiodiplodia theobromae* is a common fungus found in tropical and subtropical regions and belongs to the family Botryosphaeriaceae. Several reports confirmed the diverse array of bioactive low molecular weight compounds produced by *L. theobromae* cultures (Salvatore et al. 2020, and references therein).

Here, spores of *A. sydowii*, *A. flavus* and *L. theobromae* were separately subject to gamma radiation at several doses to study the effect of exposure gamma rays on the antifeedant and larvicidal activities of the three strains. Our data indicated that the most proper dose for maximum values of the recorded antifeedant and larvicidal activities for the three strains was at 1000 Gy. Similarly, the same irradiation dose successfully intensified the antimicrobial, antioxidant, and anticancer activities from extract of the same strains *A. sydowii*, *A. flavus* (El-Sayed et al. 2022a). Also, the same irradiation dose improved production of the cardiac glycoside digoxin to a five-fold increase by *Epicoccum nigrum* (El-Sayed et al.  2020b). Moreover, the same effect of exposure to gamma rays on the survival and the fungal growth was observed by previous reports (Awan et al. 2011; El-Sayed and Zaki 2022; El-Sayed et al. 2019a; b; c). Previous reports concluded the effect of gamma rays as an energetic ionizing radiation in induction of mutations in the exposed cells (Thacker 1999) through the repair mechanism in genes of the DNA of the irradiated cells (Ismaiel et al. 2014, 2015; Zaki et al. 2020; Zaki and El-Sayed 2021). Generally, exposure to physical mutagens such as gamma rays is a successful strategy to improve microbial strains by developing overproducers (Parekh et al. 2000) with intensified production rates (El-Sayed et al.  2020a, c; Zaki et al. 2020) with industrial applications thereby effectively lowering the overall cost of the process, as supported by several reports (Anwar et al. 2022; El-Sayed et al. 2020d, e, f; El-Sayed 2021; El-Sayed et al. 2022b; c).

In the current study, the methylene chloride extract was subjected to thin layer chromatography (TLC) and examined under UV light radiation. Separated bands from TLC plates for each fungal strain were tested for their antifeedant and larvicidal potentials. The active bands were then subjected to GC-MS analysis to identify the active compounds responsible for the antifeedant and larvicidal activities. The identification was accomplished using computer search user-generated reference libraries, incorporating mass spectra. The identified compounds PENITREM A (from *A. sydowii* and *A. flavus*), 1,3,5,8-tetramethyl-4,6-diethyl-7-[2-(methoxycarbonyl)ethyl]porphyrin (from *A. sydowii*), 2,7,12,17-Tetramethyl-3,5:8,10:13,15:18,20-tetrakis(2,2-dimethylpropano)porphyrin (from *A. flavus*), N-Diethyl-3-nitrobenzamide and Diisooctyl-phthalate (from *L. theobromae*). In the literature, Penitrems are well known toxic fungal metabolites produced by several fungal species (Boguś et al. 2021). It is capable of eliciting tremors in vertebrate animals and showed insecticidal activity against *Bombyx mori*, *Heliothis zea*, and *Spodoptera frugiperda* (González et al. 2003). Meanwhile, porphyrins are a group of heterocyclic macrocycle organic compounds with diverse biotechnological applications (Alves et al. 2015) include their successful use as insecticides (Amor and Jori 2000). Interestingly, N-Diethyl-3-nitrobenzamide, from *L. theobromae* culture, is a derivative of the most extensively used chemical repellent, N,N-diethyl-3-methylbenzamide (DEET) displayed repellency to a wide range of insects, including kissing bug, fruit flies, mosquitos, the tropical bed bug and the common bed bug (Syed and Leal 2008; Terriquez et al. 2013; Liu et al. 2017). Also, the Diisooctyl-phthalate from *L. theobromae* culture is a phthalate derivative. It possess significant mosquito larvicidal activity attributed to inhibition of acetylcholinesterase enzyme activity (Huang et al. 2021). Moreover, synthetic diethyl phthalate (Xu and He 2010) and dimethyl phthalate (Brown and Hebert 1997) have been used as active ingredients in insect repellents.

In summary, three promising endophytic fungal strains with antifeedant and larvicidal potentials against the 3rd instar *Spodoptera littoralis* larvae were reported. These strains were *A. sydowii*, *A. flavus*, and *L. theobromae*. The antifeedant and larvicidal activities of the three strains were intensified following exposure of the fungal spores to gamma rays. Extracts of the three strains were fractionated by TLC and active fractions was analyzed by GC-Mass. The present study recommends fungal endophytes as promising sources of antifeedants and larvicides which open the way to their use in several agricultural applications.

## Data Availability

All data generated or analyzed during this study are included in this published article.
